# A Silent Epidemic: Prevalence and Social Determinants of Cognitive Impairment Among Older Adults in South India

**DOI:** 10.1192/bjo.2026.11211

**Published:** 2026-06-30

**Authors:** Amutha Govindaraju, Arokia Antonysamy, Guganathan Regunathan, Rajeshwaran Karunanithi, Asharani Mathivanan

**Affiliations:** 1 Theni Medical College, Theni, India; 2 UnitedKingdom

## Abstract

**Aims:**

Population ageing in India is accelerating, accompanied by a rising burden of neurocognitive disorders that contribute substantially to disability, healthcare utilisation, and loss of independence. Cognitive impairment (CI), particularly in its mild and early stages, remains under-detected in low- and middle-income settings due to limited screening, low literacy levels, and restricted access to specialist services. Rural and socioeconomically disadvantaged populations are especially vulnerable, yet robust multicentric data from South India remain scarce. This study aimed to estimate the prevalence of cognitive impairment across specific cognitive domains among older adults in South India and to examine its association with socio-demographic and clinical variables.

**Methods:**

A multicentric cross-sectional survey was conducted across selected centres in South India, enrolling 122 participants aged 60 years and above. Data were collected from medical outpatient clinic attendees, using a structured proforma capturing age, gender, education, employment status, socioeconomic status (SES), medical comorbidities, and alcohol/substance use. Cognitive functioning was assessed using the Montreal Cognitive Assessment (MoCA), a validated and widely used screening instrument for detecting cognitive impairment. Cognitive impairment was categorised into mild, moderate and severe, based on established cut-offs. Statistical analyses were performed to identify significant correlates of cognitive impairment.

**Results:**

The study population was predominantly male (68.9%), below 65 years of age (58.2%), and residing in rural areas (79.5%). The overall prevalence of cognitive impairment was strikingly high at 77.9%. Of those identified with impairment, 59.8% had mild cognitive impairment, indicating a substantial burden of potentially reversible or modifiable cognitive decline. Cognitive impairment showed significant associations with increasing age, lower educational attainment, unemployment or engagement in unskilled labour, and lower socioeconomic status. In contrast, no statistically significant associations were observed with gender, rural versus urban residence, medical comorbidities, or alcohol/substance use.

**Conclusion:**

This study demonstrates a markedly high prevalence of cognitive impairment among older adults in South India, with a large proportion experiencing early or mild cognitive deficits that are likely undetected in routine care. The strong association with social and educational disadvantage highlights cognitive impairment as not only a medical condition but also a social determinant, driving health inequity. These findings emphasise the urgent need to integrate routine cognitive screening into primary healthcare and geriatric services. Early identification and timely intervention may help delay progression, reduce disability, and preserve functional independence, thereby improving quality of life for India’s rapidly ageing population.

